# Supplementary material for: Modeling glioblastoma heterogeneity as a dynamic network of cell states
Source: Mol Syst Biol. 2021 Sep 16;17(9):e10105. doi: 10.15252/msb.202010105 (PMC8444284; doi:10.15252/msb.202010105)
Supplement: Supplementary file 6 — Source Data for Figure 5 [file MSB-17-e10105-s004.zip › Figure5A_sourcedata/GSEA_3017/hallmarks_stateC.GseaPreranked.1621934520804/HALLMARK_APOPTOSIS.html]

Details for gene set HALLMARK\_APOPTOSIS[GSEA]

|  || Dataset | state13017 |
| Phenotype | NoPhenotypeAvailable |
| Upregulated in class | na\_neg |
| GeneSet | HALLMARK\_APOPTOSIS |
| Enrichment Score (ES) | -0.3810362 |
| Normalized Enrichment Score (NES) | -1.6135491 |
| Nominal p-value | 0.035772357 |
| FDR q-value | 0.06820116 |
| FWER p-Value | 0.385 |
Table: GSEA Results Summary

  

Fig 1: Enrichment plot: HALLMARK\_APOPTOSIS      
 Profile of the Running ES Score & Positions of GeneSet Members on the Rank Ordered List

  

| PROBE | GENE SYMBOL | GENE\_TITLE | RANK IN GENE LIST | RANK METRIC SCORE | RUNNING ES | CORE ENRICHMENT || 1 | HMGB2 |  |  | 20 | 0.534 | 0.0264 | No |
| 2 | TOP2A |  |  | 52 | 0.426 | 0.0213 | No |
| 3 | BRCA1 |  |  | 54 | 0.410 | 0.0665 | No |
| 4 | CCND2 |  |  | 149 | 0.288 | -0.0635 | No |
| 5 | SAT1 |  |  | 241 | -0.262 | -0.1913 | No |
| 6 | TGFB2 |  |  | 285 | -0.272 | -0.2347 | No |
| 7 | MMP2 |  |  | 340 | -0.291 | -0.2950 | No |
| 8 | LMNA |  |  | 376 | -0.308 | -0.3204 | No |
| 9 | RHOB |  |  | 391 | -0.316 | -0.3086 | No |
| 10 | TIMP1 |  |  | 399 | -0.320 | -0.2841 | No |
| 11 | GADD45A |  |  | 405 | -0.323 | -0.2557 | No |
| 12 | LGALS3 |  |  | 471 | -0.374 | -0.3256 | No |
| 13 | JUN |  |  | 503 | -0.425 | -0.3308 | Yes |
| 14 | IFITM3 |  |  | 533 | -0.477 | -0.3265 | Yes |
| 15 | TNFRSF12A |  |  | 550 | -0.519 | -0.2949 | Yes |
| 16 | TIMP3 |  |  | 553 | -0.530 | -0.2378 | Yes |
| 17 | PLAT |  |  | 556 | -0.538 | -0.1798 | Yes |
| 18 | CD44 |  |  | 575 | -0.659 | -0.1356 | Yes |
| 19 | CLU |  |  | 581 | -0.690 | -0.0655 | Yes |
| 20 | EMP1 |  |  | 589 | -0.785 | 0.0121 | Yes |
Table: GSEA details [plain text format]

  

Fig 2: HALLMARK\_APOPTOSIS: Random ES distribution      
 Gene set null distribution of ES for **HALLMARK\_APOPTOSIS**
